# LLL12, a novel small inhibitor targeting STAT3 for hepatocellular carcinoma therapy

**DOI:** 10.18632/oncotarget.3458

**Published:** 2015-03-26

**Authors:** Mingxin Zuo, Chenglong Li, Jiayuh Lin, Milind Javle

**Affiliations:** ^1^ Department of Gastrointestinal Medical Oncology, The University of Texas MD Anderson Cancer Center, Houston, TX 77030, USA; ^2^ Division of Medicinal Chemistry and Pharmacognosy, College of Pharmacy, The Ohio State University, Columbus, OH 43210, USA; ^3^ Center for Childhood Cancer, The Research Institute at Nationwide Children's Hospital, Department of Pediatrics, College of Medicine, The Ohio State University, Columbus, Ohio 43205, USA

**Keywords:** STAT3, hepatocellular carcinoma, LLL12, small molecular inhibitor

## Abstract

The constitutive activation of signal transducer and activator of transcription 3 (STAT3) is frequently detected in clinical incidences of hepatocellular carcinoma (HCC) but not in normal human hepatocytes. STAT3 signaling plays pivotal roles in angiogenesis, survival, metastasis, and growth of HCC. Recent evidence suggests that the blockade of aberrant STAT3 pathways can be exploited as a therapeutic strategy for HCC. We have developed the novel small molecular STAT3 inhibitor LLL12 on the basis of curcumin structure using computer-aided rational design. LLL12 has shown antitumor activity in various solid tumors including breast, brain, pancreatic cancer, and glioblastoma *in vitro* and *in vivo*. In this study, we hypothesized LLL12 inhibits STAT3 phosphorylation at tyrosine 705 (Y705) in HCC and show antitumor activity in HCC *in vitro* and *in vivo*. Our results show that LLL12 selectively inhibited HCC cell proliferation and induced apoptosis in SNU387, SNU398, SNU449, and Hep3B HCC cells *in vitro*. Furthermore, LLL12 at 5 mg/kg/day significantly inhibited the growth of SNU398 xenografts in nude mice. Collectively, our results indicate that LLL12 could be used to target STAT3 for the effective prevention or treatment of HCC.

## INTRODUCTION

Liver cancer is the sixth most common malignancy and the third-leading cause of cancer death worldwide. Hepatocellular carcinoma (HCC) accounts for 90% of all liver cancers. In the United States, the estimated new liver cancer cases and deaths in 2014 account for 33,190 and 23,000, respectively [1]. HCC is a complex and heterogeneous disease with various genomic alterations. Several of aberrant activation of cell signaling cascades such as the IL6/JAK/Stat3, EGFR, Ras/ERK, PI3K/mTOR, and Wnt signaling pathways have been found in HCC [2–6]. Currently, there is no effective therapy for HCC, Surgical excision by partial or total hepatectomy represents the only potentially curative therapy for HCC, but many patients would be in an advanced stage of disease at initial diagnosis. Most of these patients aren't candidates for surgery. Therefore, there is a pressing need for the development of new approaches in HCC therapy.

Recently, it was reported that STAT family proteins, especially STAT3 play a crucial role in the initiation of various cancer transformation and progression. STAT3 was initially discovered as an acute-phase response protein, suggesting its link to inflammation. Also, most proinflammatory agents have been shown to activate STAT3 [7, 8]. Mounting clinical and epidemiologic evidence suggests that chronic hepatitis viral infections, cirrhosis, are related to HCC [9, 10]. Inflammation of the liver increases HCC risk by promoting liver cirrhosis; indeed, the majority of HCC cases begin with inflammation that leads to cirrhosis and then to HCC [11]. Chronic inflammation and aberrant activity of STAT3 cell signaling pathway play important roles in hepatocarcinogenesis, for all these reasons, STAT3 represents an attractive target in HCC therapy. Thus, a blockade of aberrant STAT3 signaling pathways can be exploited as a therapeutic strategy for HCC [5, 12–14].

**Figure 1:**
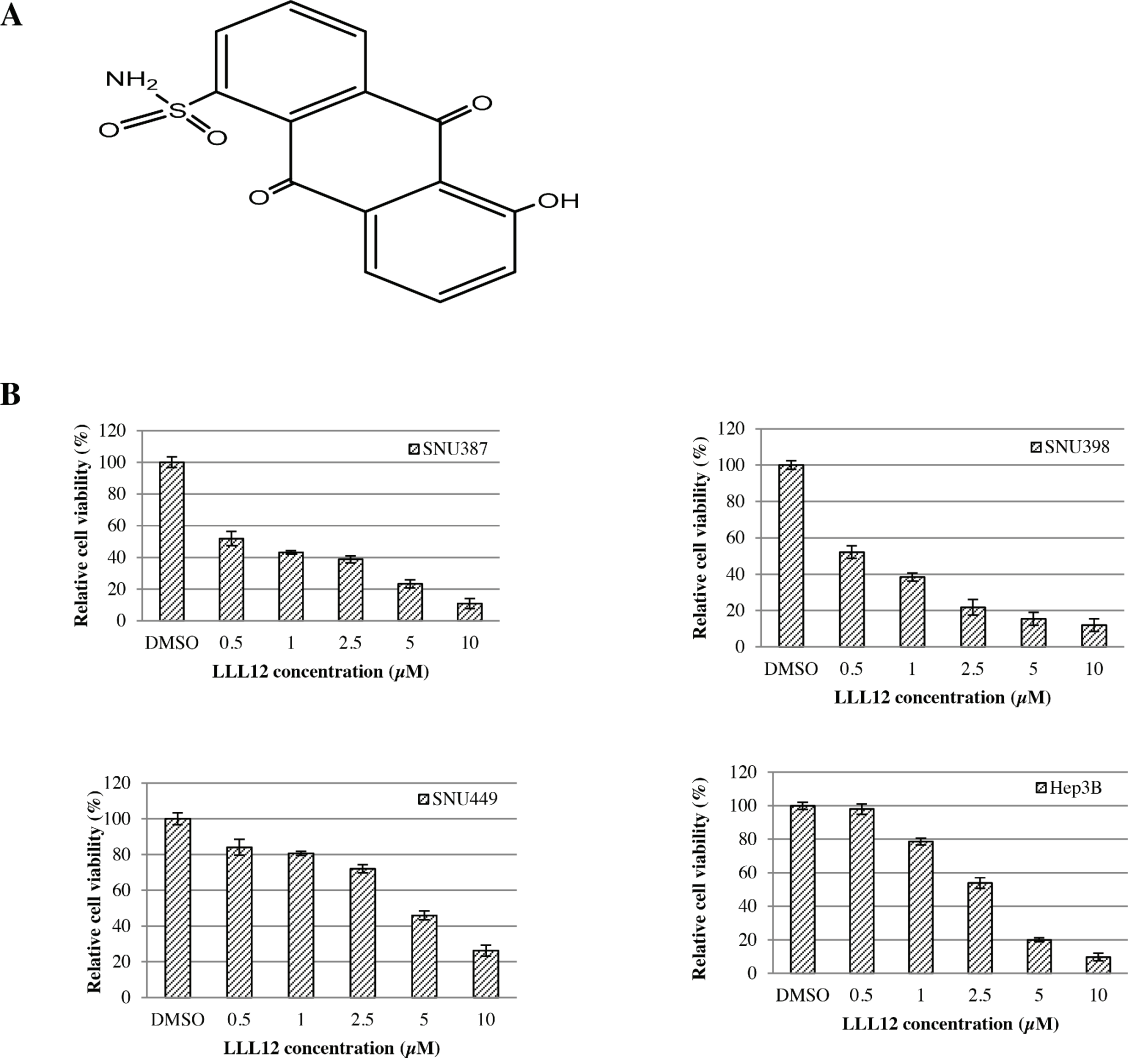
**(A) Structure of LLL12. (B)** Effects of LLL12 on the proliferation of HCC cell lines. HCC cell lines (SNU387, SNU398, SNU449, and Hep3B) were treated with DMSO or LLL12 with serial concentrations for 72 h. Proliferation was analyzed by MTT. Proliferation values are listed as percentage of DMSO control. The IC_50_ values shown are mean ± standard deviation for three separate experiments.

We have developed a novel small molecular STAT3 inhibitor, LLL12, a substituted anthraquinone, which synthesized based on curcumin structure using computer-aided rational design (Figure 1A). Several study results show curcumin has antitumor activity; however, curcumin has low solubility.

We have reported LLL12 inhibits STAT3 activity by selectively binding to its Src homology 2 domain and show potent antitumor activity in breast cancer, brain cancer, and pancreatic cancer [15–18]. In this article, we explored the antitumor activities of LLL12 on HCC *in vitro* and *in vivo*.

## RESULTS

### LLL12 inhibited growth and induced apoptosis in various human HCC cell lines

**Figure 2:**
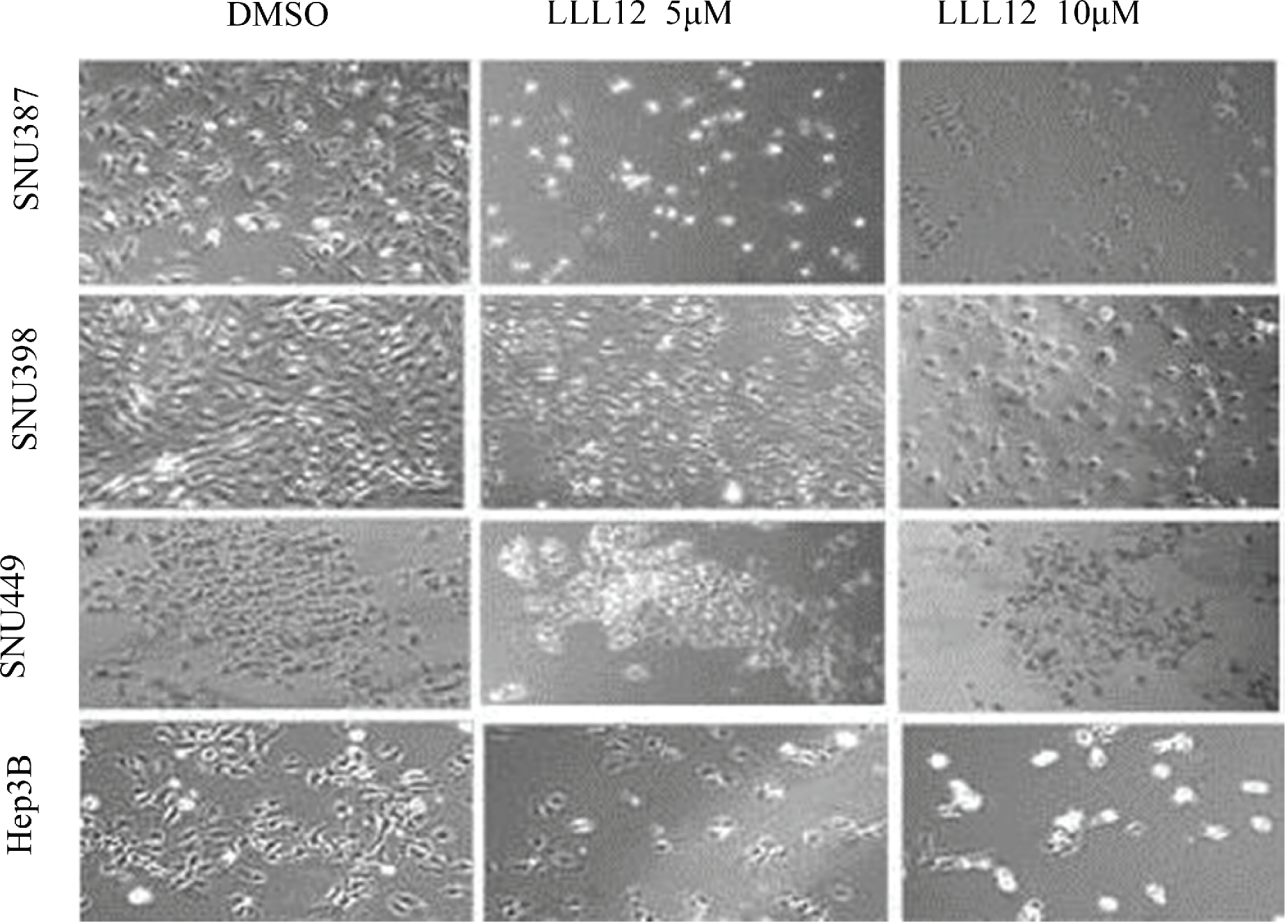
Morphologic features of LLL12-induced cell death in HCC cell lines Cell morphology was visualized using light microscopy after treatment with DMSO or with 5 μM or 10 μM LLL12 for 24 h.

To examine the cytotoxic activity of LLL12, the human HCC cell lines SNU387, SNU398, SNU449, and Hep3B were treated with LLL12 at serial concentrations and assessed by the 3-(4,5-dimethylthiazol-2-yl)-2, 5-diphenyltetrazolium bromide (MTT) assay. LLL12 significantly inhibited growth in all four cell lines in a dose-dependent fashion (Figure 1B), with 50% inhibition (IC_50_) values at 72 h of 0.84 ± 0.23 μM, 0.96 ± 0.18 μM, 4.38 ± 1.25 μM, and 2.39 ± 0.68 μM, respectively. Morphologic assays of SNU387, SNU398, SNU449, and Hep3B treated with LLL12 at 5 μM or 10 μM for 24 h showed that LLL12 inhibited cell growth and induced substantial apoptosis in most of the cell lines at 10 μM (Figure 2).

### LLL12 inhibited phosphorylation of STAT3 and induced HCC cell apoptosis

**Figure 3:**
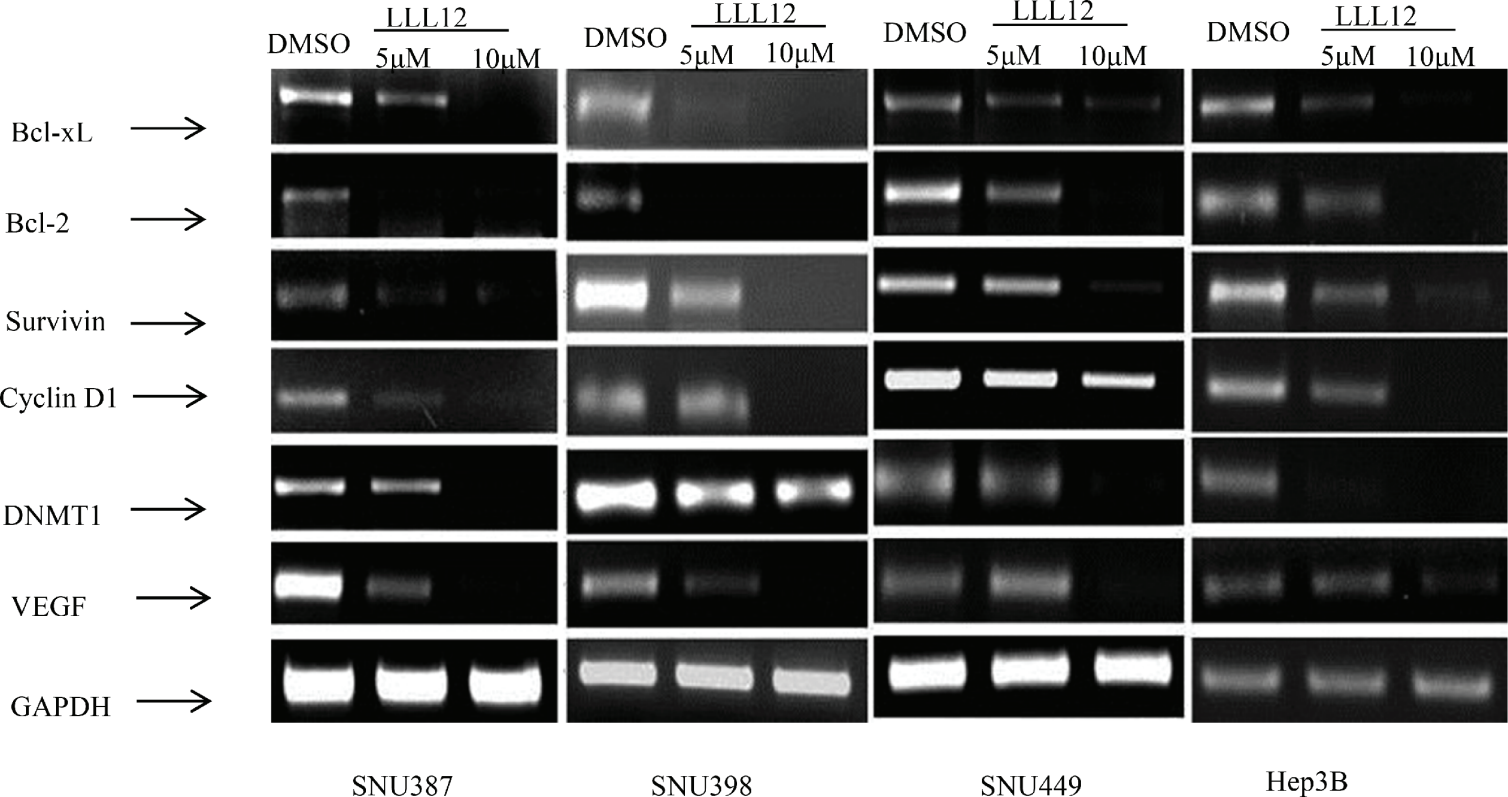
Decreased expression levels of STAT3 target genes upon treatment with LLL12 HCC cell lines were treated with DMSO or with 5 μM or 10 μM LLL12 for 24 h, and cells were collected for RNA extraction. Experiments were repeated three times.

**Figure 4:**
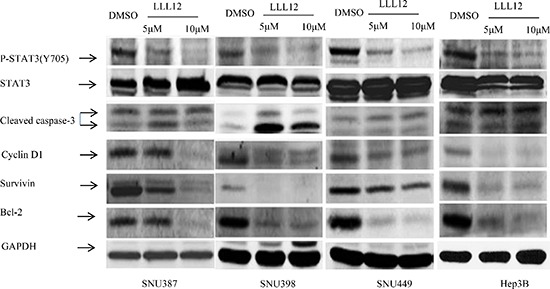
Effects of LLL12 on STAT3 target protein expressions HCC cell lines were treated with DMSO or with 5 μM or 10 μM LLL12 for 24 h. Protein lysates were generated and separated by SDS–polyacrylamide gel electrophoresis and Western blotting for pSTAT3 (Y705)–, survivin-, and apoptosis-related protein expression.

RT-PCR results showed that LLL12 modulated the expression of the cell cycle regulator gene *cyclin D1*; the anti-apoptotic gene *Bcl-2*, *Bcl-xL*, *survivin*, and *DNMT1*; and the angiogenic gene product *VEGF* (Figure 3), all of which are reportedly regulated by STAT3. The Western blot results show that LLL12 decreased phosphorylation of STAT3 protein (at Y705), decreased survivin and Bcl-2 protein expression, and increased cleaved caspase-3 protein expression (Figure 4).

### LLL12 caused the accumulation of HCC cells in the G2/M phase

**Figure 5:**
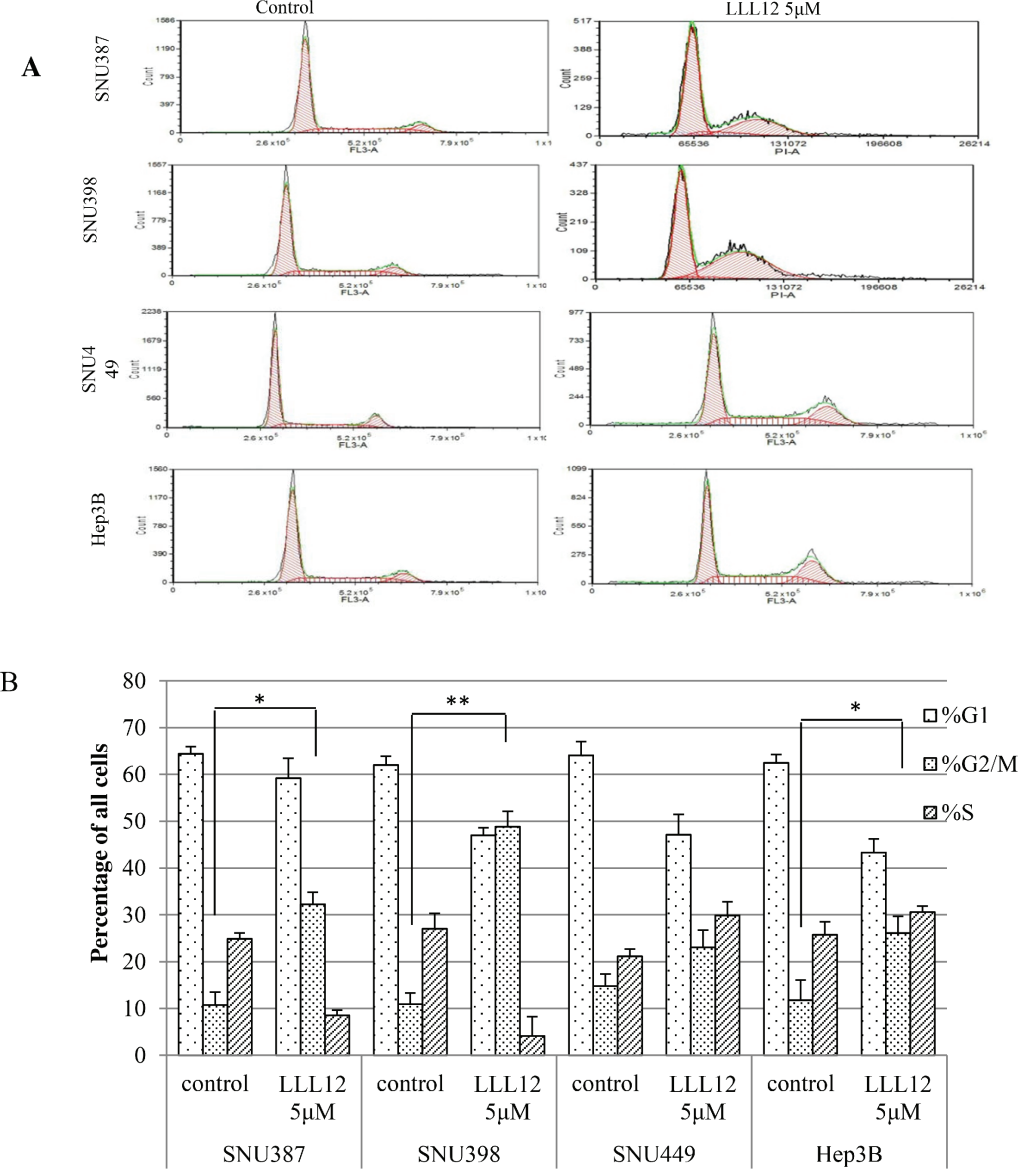
Flow cytometric analysis of the cell cycles of HCC cells **(A)** HCC cells treated with DMSO or 5 μM LLL12 for 24 h were harvested. The LLL12 treatment increased the number of HCC cells arrested in the G2 phase. **(B)** Quantitative analysis of HCC cells from three independent experiments Means ± standard deviations is shown. **P < 0.01; *P < 0.05.

The effects of LLL12 on the HCC cell cycle were examined by flow cytometry. The cells were synchronized by incubation overnight in the absence of serum and then treated with dimethyl sulfoxide (DMSO) or LLL12 (10 μM) for 24 h. The distributions of cells in each phase are shown in Figure 5. There were significant increases in cells at the G2/M phase after LLL12 exposure in SNU387, SNU398, and Hep3B cells.

### LLL12 suppressed nuclear translocation of STAT3 in SNU398 and SNU387

**Figure 6:**
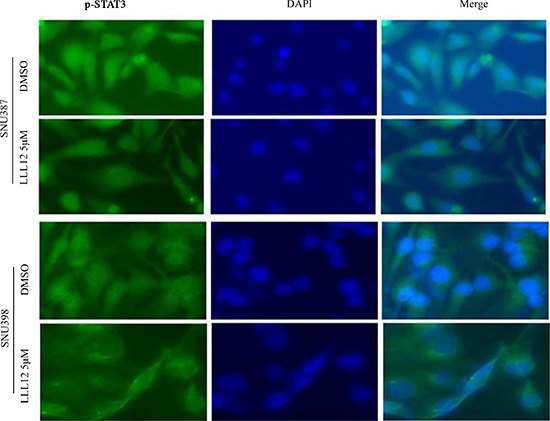
Immunofluorescence staining for p-STAT3 protein in HCC cell lines SNU387 and SNU398 cells were exposed to DMSO or LLL12 (5 μM) for 24 h. Subcellular localization and expression of STAT3 (green) was analyzed by immunofluorescent staining. Nuclei were counterstained using DAPI (blue).

Phosphorylation of STAT3 (Y705) is required for nuclear translocation, which plays central roles in regulating STAT3 downstream target genes. To determine whether LLL12 can suppress nuclear translocation of STAT3, we performed an immunofluorescence assay using monoclonal antibodies against p-STAT3 protein. Images of fluorescence from cells stained with fluorescein isothiocyanate (FITC)-4′,6-diamidino-2-phenylindole (DAPI) show that LLL12 inhibits STAT3 phosphorylation and decreases the amount of p-STAT3 in the nucleus in SNU398 and SNU387 cells stained with a monoclonal anticytokeratin FITC-conjugated secondary antibody (Figure 6). However, no significantly inhibitive effects of LLL12 on nuclear translocation of STAT3 were observed in SNU449 and Hep3B cells (data not shown), possibly because SNU449 and Hep3B cells are more resistant to LLL12 than the other HCC cell lines.

### LLL12 suppressed the growth of SNU398 xenograft tumors *in vivo*

**Figure 7:**
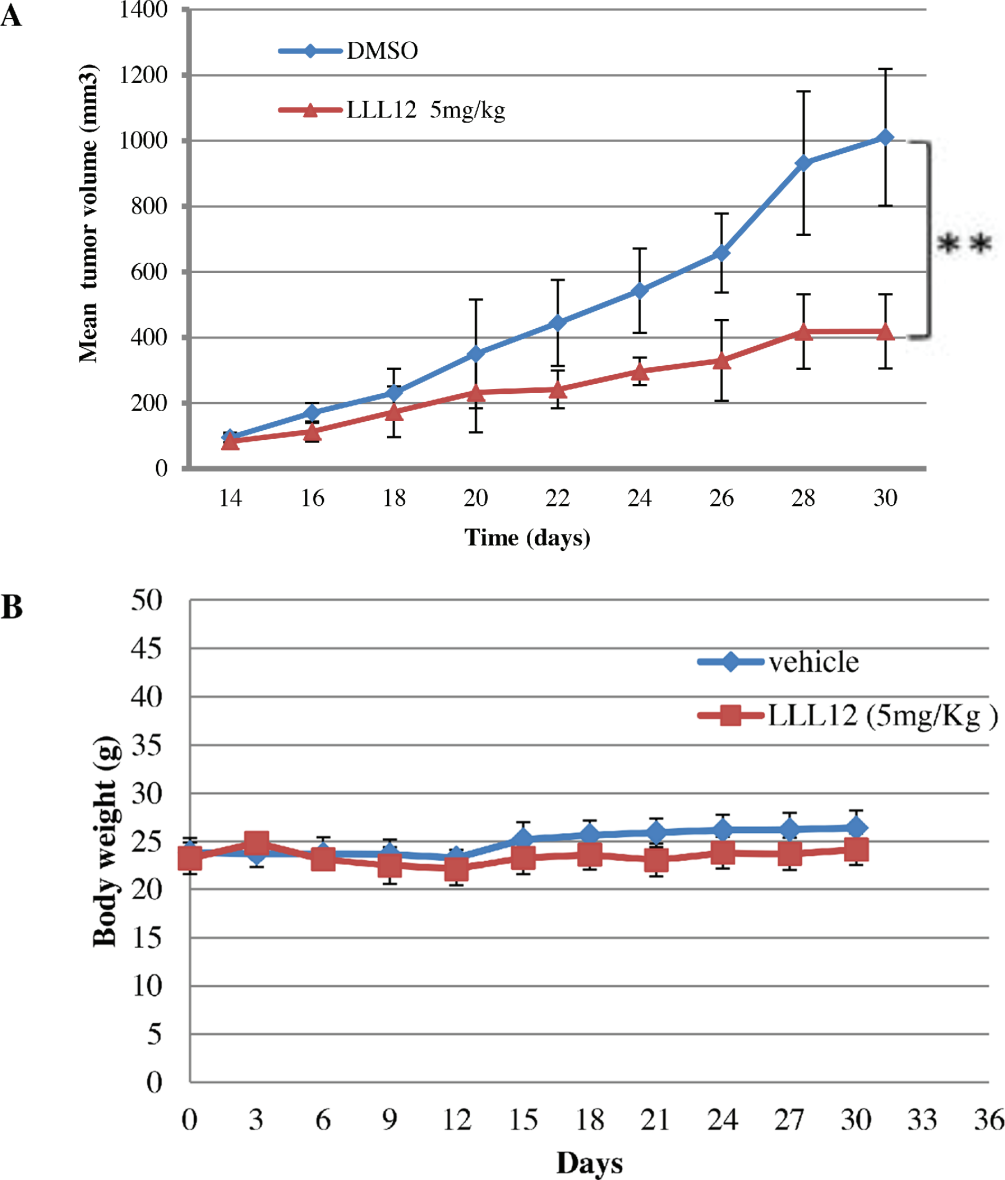
Effects of LLL12 on SNU398 xenografts **(A)** Nude mice were subcutaneously injected with 5 × 10^6^ SNU398 cells, and then treated with a vehicle or LLL12 (5 mg/kg/day) for 17 days. The tumor volume in the treatment group was significantly smaller than that in the control group, as determined by Student *t*-tests (**P < 0.01). **(B)** Body weights of nude mice treated with LLL12 or vehicle have no significant difference over a period of 30 days. Data in (A) and (B) are presented as the mean ± standard deviation for eight mice each group.

To examine the antitumor activity of LLL12 *in vivo*, xenograft experiments were performed in nude mice by implanting 5 × 10^6^ SNU398 cells via subcutaneous injection into each mouse and treating these mice bearing tumors with either LLL12 (5 mg/kg) or with a vehicle (DMSO) by intraperitoneal injection every day for 17 days. LLL12 significantly inhibited tumor growth compared with the control vehicle (Figure 7A). The body weights of the mice in both groups have no significant difference over the course of treatment (Figure 7B).

## DISCUSSION

HCC is the major of liver cancer and the incidence of primary liver cancer is increasing in several developed countries and the increase will likely continue for some decades. Many genetic alterations and critical molecular signaling pathways have been identified as contributing to HCC development and progression. These pathways include PI3K/Akt/mTOR, Wnt/β-catenin, NF-κB and STAT3 signaling pathway. However, the molecular pathogenesis of HCC is not well-understood. As a result, there are no effective mechanism-based therapies for HCC. In STAT3 knockout mouse models, it has been shown that STAT3 is required for tumorigenesis in mouse intestine, skin and liver [19–21]. As compelling data continue to accumulate STAT3 has linked inflammation and oncogenes and phosphorylation of STAT3 at Y705 is also frequently associated with a poor prognosis. STAT3 has become an attractive target for treatment and prevention of HCC.

LLL12, a small molecular inhibitor, was synthesized according to curcumin structure. Computer models with docking simulation showed that LLL12 binds directly to phosphorylated Y705 binding site of monomers [16, 22] and LLL12 does not inhibit STAT3 upstream kinase like JAK1, JAK2 and TYK2. We found that LLL12 inhibited the SNU387, SNU398, SNU449, and Hep3B human HCC cell growth, arrested HCC cells in the G2/M phase and induced these cell apoptosis *in vitro*. Our immunofluorostaining results show LLL12 can effectively inhibit p-STAT3 nuclear transfer in SNU 398 and SNU 387 cells, but the inhibition of p-STAT3 nuclear localization was not observed in Hep3B and SNU449 cancer cells. LLL12 decreased phosphor-STAT3 (Y705), cyclin D and survivin protein expression in SNU398, SNU387 and Hep3B cells. However, we did not observer LLL12 decreased cyclinD1 and survivin protein expression in SNU449 cells. These results show SNU398 cells are more sensitive to LLL12 than SNU449 cells which are consistent with the cell proliferative results that the IC_50_ value of LLL12 on SNU449 cells is higher than on other HCC cell lines. In SNU398 xenograft model, LLL12 at 5mg/kg can significantly inhibited tumor growth. The various effects of LLL12 on these cell lines may be related to the heterogeneous of HCC. However, STAT3 pathway has interaction with many other cell signaling pathways like NF-κB, PI3K/Akt/mTOR pathway. So, the combination of LLL12 with other multiple kinase inhibitors such as sorafenib is deserved to be tested on HCC cell lines in the future. On the other sides, Although, LLL12 have more potent antitumor activity than curcumin, it still has relative low solubility. So, how to increase LLL12 solubility may be another strategy to improve the antitumor activity on HCC.

In conclusion, LLL12 show significant inhibitory effects on STAT3 phosphorylation and activation, nuclear accumulation, and transcriptional activity in HCC cell lines. LLL12 also show significant antitumor activity in nude mice implanted with SNU398 cells. LLL12 may provide a novel strategy for HCC prevention and therapy

## MATERIALS AND METHODS

### Materials

LLL12 was provided by the Ohio State University College of Pharmacy and dissolved in DMSO. For the MTT assay (Research Products International, Mount Prospect, IL), the following antibodies were used: phospho-Stat3 (Y705) antibody, Stat3 antibody, cleaved caspase-3 (Asp175) antibody, cyclin D1 antibody, survivin antibody, and biotinylated glyceraldehyde 3-phosphate dehydrogenase (GAPDH) (14C10) rabbit antibody (Cell Signaling Technology, Beverly, MA).

### Cell cultures

The HCC cell lines SNU387, SNU398, SNU449, and Hep3B were obtained from the ATCC (Manassas, VA, USA). SNU398 and SNU449 cells were cultured with Roswell Park Memorial Institute 1640 medium with 10% heat-inactivated fetal bovine serum, SNU387 cells were maintained in Roswell Park Memorial Institute 1640 medium with 10% fetal bovine serum, and Hep3B cells were cultured with ATCC-formulated Eagle's minimum essential medium with 10% fetal bovine serum. One hundred units of penicillin and streptomycin were added to each cell culture media.

### MTT assay

The MTT assay provides a quantitative determination of viable cells. We seeded 3 × 10^3^ cells in 96-well microplates in complete culture medium in the absence or presence of increasing serial dosages of LLL12 compound in triplicate. Cells were cultured at 37°C for 72 h, and the number of viable cells was measured by adding 50 μl of 5 mg/ml MTT solution per well. After 4 h, the formazan crystals were dissolved by adding 100 μl solution [23]. The absorbance was read at 595 nm with an enzyme-linked immunosorbent assay reader. IC_50_ values were calculated by SigmaPlot 9.0 software (Systat Software, Inc., San Jose, CA, USA).

### Immunofluorescence assay

To determine the effect of LLL12 on the protein levels of STAT3 in SNU398 SNU387, SNU449, and Hep3B cells. We performed immunofluorescence staining using the p-STAT3 antibody. Briefly, we seeded the SNU398 SNU387, SNU449, and Hep3B cells in six-well plates, treated the cells with LLL12 at the 5 μM for 24 h, and removed the media and washed the cells three times with phosphate-buffered saline (PBS) solution. The cells were then fixed with pre-cooled menthol for 15 min. After washed with PBS, the coverslips were incubated with the primary antibody (1:100) in PBS with 3% goat serum albumin overnight at 4°C, washed with PBS, and then incubated with FITC-conjugated secondary antibodies (1:100) in the dark at room temperature for 1 h. For nuclear staining, the cells were stained with DAPI 1.5 μg/ml for 5 min before examination. A fluorescence microscope was used to visualize the cells stained with p-STAT3 antibody and/or DAPI.

### Reverse transcription polymerase chain reaction (RT-PCR)

Total RNA was extracted from cells from all four HCC lines treated with LLL12 (5 μM, or 10 μM) or DMSO for 24 h using RNeasy Kits (QIAGEN, Valencia, CA, USA). Reverse transcription was carried out at 42°C for 50 minutes using oligo(dT) sequences as primers. Two microliters of this reverse transcription product was then used for RT-PCR. RT-PCR was carried out in 25 μl total volume of reaction buffer. The oligonucleotides used for amplification of the cDNA were sense and antisense, which were synthesized by Integrated DNA Technologies, Inc. (Coralville, IA, USA) (Table 1). After the initial denaturation of 5 min at 95°C, RT-PCR was performed for 25 cycles in an Eppendorf Mastercycler Gradient Thermal Cycler (Eppendorf, NY, USA). Ten microliters of each RT-PCR product were run on a 2% (w/v) agarose gel containing ethidium bromide. Gel images were captured using Chemigenius Gel Documentation System (Syngene, Cambridge, UK).

**Table 1:** Primer sequences used in RT-PCR assay

Gene	Primers	Size
**Bcl-xL**	Forward: 5′-TTGGACAATGGACTGGTTGA-3′ Reverse: 5′-GTAGAGTGGATGGTCAGTG-3′	765
**Bcl-2**	Forward: 5′-TCTTTGAGTTCGGTGGGGTC-3′ Reverse: 5′-TGCATATTTGTTTGGGGCAGG-3′	304
**Survivin**	Forward: 5′-ACCAGGTGAGAAGTGAGGGA-3′ Reverse: 5′-AACAGTAGAGGAGCCAGGGA-3′	309
**Cyclin D1**	Forward: 5′-GCTGGAGCCCGTGAAAAAGA-3′ Reverse: 5′-CTCCGCCTCTGGCATTTTG-3′	247
**VEGF**	Forward: 5′-GAGTACCCTGATGAGATCGAG-3′ Reverse: 5′-TCACCGCCTCGGCTTGTCACA-3′	459
**DNMT1**	Forward: 5′-GTGGGGGACTGTGTCTCTGT-3′ Reverse: 5′-TGAAAGCTGCATGTCCTCAC-3′	204
**GAPDH**	Forward: 5′ CGCTCTCTGCTCCTCCTGTT 3′ Reverse: 5′ CCATGGTGTCTGAGCGATGT 3′	81

### Cell cycle assay by flow cytometry

Cells from all four HCC lines were seeded in 100 × 20 mm dishes. Cells were synchronized by incubation overnight in the absence of serum before DMSO or LLL12 (5 μM) was added, and then the cells were incubated for 24 h. Then, 1–2 × 10^6^ single cells were collected and washed with cold PBS, fixed with precooled 70% ethanol, incubated on ice a minimum of 45 min. The cells were then centrifuged, carefully aspirated from the supernatant, and then these cells were re-suspended in propidium iodide (final concentration, 40 μg/ml) and RNase A (final concentration, 100 μg/ml) solution at a final cell density of 0.5 × 10^6^ cells/ml. The suspension was incubated at 37°C for 30 min prior to analysis by flow cytometry.

### Western blot analysis

Cells from all four HCC lines were plated in dishes 100 mm in diameter, and after the cells had grown in a monolayer to 70% confluence, the medium was replaced with fresh medium containing DMSO or containing LLL12 (5 μM or 10 μM). The cells were then cultured for 24 h before they were harvested for Western blot analysis. The cells were washed twice with cold PBS and lysed on ice for 10 min with radioimmunoprecipitation assay buffer (10 mM Tris, pH 8.0), 150 mM NaCl, 1% sodium deoxycholate, 0.1% sodium dodecyl sulfate (SDS), 1% Triton X-100, 10 μg/ml leupeptin, 10 μg/ml aprotinin, 1 mM phenylmethylsulfonyl fluoride. Lysates were centrifuged at 13,000 rpm at 4°C for 20 min. The protein concentration of supernatant was determined with Bicinchoninic acid protein assay kit (Thermo Scientific, IL, USA), the lysate proteins were mixed with 4 × SDS gel sample buffer and separated with 10% SDS–polyacrylamide gel electrophoresis. Gel proteins were transferred by Western blot to Hybond polyvinylidene difluoride membrane. Before undergoing blotting with primary antibodies, the membrane was blocked with 5% milk solution for 1 h and then incubated with a 1:1000 dilution of primary antibody for 2 h at room temperature, washed three times with TBST for 5 min, blotted with a 1:1000 dilution of secondary antibody, washed three times, and incubated with tertiary antibody. The membranes were scanned with Scanner STORM 860 (Amersham Biosciences, Amersham, UK).

### Xenograft transplation and *in vivo* tumor studies

Six-week-old female athymic nude mice were purchased from Harlan Laboratories (Indianapolis, IN, USA) and maintained in institutional animal facilities approved by the American Association for Accreditation of Laboratory Animal Care. The mice were injected subcutaneously in the left flank area with 5 × 10^6^ SNU398 human HCC cells. After the tumors reached at 100 to 150 mm^3^ in size, the mice were randomized into control and treatment groups. The mice were then given LLL12 at 5 mg/kg or a vehicle (DMSO) intraperitoneal injection every day. Tumor volume (*V *) was calculated according to the formula *V* = 0.5 × *a*^2^ × *b*, where *a* is the smallest superficial diameter and *b* is the largest superficial diameter. The Student *t*-test was used to analyze *in vivo* growth patterns of the tumors and of total body weights.

### Statistical analysis

All experiments were performed two to three times. Results are expressed as means ± standard deviation. Statistical comparisons of results from the treatment and control groups were done using the Student *t*-test and one-way analysis of variance. A *P* value of less than 0.05 was considered statistically significant.
